# A dataset of small molecules triggering transcriptional and translational cellular responses

**DOI:** 10.1016/j.dib.2018.02.061

**Published:** 2018-02-27

**Authors:** Mathilde Koch, Amir Pandi, Baudoin Delépine, Jean-Loup Faulon

**Affiliations:** a Micalis Institute, INRA, AgroParisTech, Université Paris-Saclay, 78350 Jouy-en-Josas, France; b UMR 8030 Genomics Metabolics, Systems and Synthetic Biology Lab, CEA, CNRS, University of Evry-val-d’Essonne, University Paris-Saclay, Évry, France; c CEA, DRF, IG, Genoscope, Évry 91000, France; d SYNBIOCHEM Centre, Manchester Institute of Biotechnology, University of Manchester, 131 Princess Street, Manchester M1 7DN, UK

## Abstract

The aim of this dataset is to identify and collect compounds that are known for being detectable by a living cell, through the action of a genetically encoded biosensor and is centred on bacterial transcription factors. Such a dataset should open the possibility to consider a wide range of applications in synthetic biology. The reader will find in this dataset the name of the compounds, their InChI (molecular structure), the publication where the detection was reported, the organism in which this was detected or engineered, the type of detection and experiment that was performed as well as the name of the biosensor. A comment field is also provided that explains why the compound was included in the dataset, based on quotes from the reference publication or the database it was extracted from. Manual curation of *ACS Synthetic Biology* abstracts (Volumes 1 to 6 and Volume 7 issue 1) was performed as well as extraction from the following databases: Bionemo v6.0 (Carbajosa et al., 2009) [1], RegTransbase r20120406 (Cipriano et al., 2013) [2], RegulonDB v9.0 (Gama-Castro et al., 2016) [3], RegPrecise v4.0 (Novichkov et al., 2013) [4] and Sigmol v20180122 (Rajput et al., 2016) [5].

**Specifications Table**

**Table:** 

Subject area	*Biology*
More specific subject area	*Synthetic biology*
Type of data	*Table*
How data was acquired	*Database extraction from Bionemo v6.0, RegTransbase r20120406, RegulonDB v9.0, RegPrecise v4.0 and Sigmol v20180122 as well as manual curation ACS Synthetic Biology abstracts (Volumes 1 to 6 and Volume 7 issue 1)*
Data format	*Analysed*
Experimental factors	*Not applicable*
Experimental features	*Not applicable*
Data source location	https://github.com/brsynth/detectable_metabolites
Data accessibility	*Data is with this article and on GitHub at*https://github.com/brsynth/detectable_metabolites

**Value of the data**

• This dataset provides a basis for the development of new biosensing circuits for synthetic biology and metabolic engineering applications, e.g. the design of whole-cell biosensor, high-throughput screening experiments, dynamic regulation of metabolic pathways, transcription factor engineering or creation of sensing-enabling pathways.
• This dataset provides a unique source of a broad number of compounds that can be detected and acted upon by a cell, increasing the possibility of orthogonal circuit design from the few usual compounds used in those applications.
• The manually curated section provides information on where the biosensor has been first reported and successfully used, enabling the reader to select trustworthy information for his application of choice.
• Detectable compounds can be searched by both by name and chemical similarity.
• This dataset is an update of [10.6084/m9.figshare.3144715.v1].

## 1 Data

The aim of this dataset is to identify and collect compounds that are known for being detectable by a living cell, through the action of a genetically encoded biosensor and is centred on bacterial transcription factors. The dataset should allow the synthetic biology community to consider a wide range of applications. The reader will find in this dataset the name of the compounds, their InChI (molecular structure), the publication where the detection was reported, the organism in which this was detected or engineered, the type of detection and experiment that was performed as well as the name of the biosensor. A comment field is also provided that explains why the compound was included in the dataset, based on quotes from the reference publication or the database it was extracted from. Manual curation of *ACS Synthetic Biology* abstracts (Volumes 1 to 6 and Volume 7 issue 1) was performed as well as extraction from the following databases: Bionemo v6.0 [1], RegTransbase r20120406 [2], RegulonDB v9.0 [3], RegPrecise v4.0 [4] and Sigmol v20180122 [5].

This dataset is available online on GitHub to allow for further updates as well as community contributions.

## 2 Experimental design, materials and methods

• *Manual curation of ACS Synthetic Biology (Volume 1–6 and Volume 7 issue 1):*
  All abstracts of *ACS Synthetic Biology* (Volume 1–6 and Volume 7 issue 1) were read and information relevant to this dataset was extracted from those abstracts. The aim of this manual curation was to establish a list of detectable compounds whose detection method was already successfully implemented in a synthetic circuit, providing a good basis for further implementation for synthetic biologists.
• *Bionemo v6.0* [1]:
  The SQL request used to create this dataset is:
  SELECT DISTINCT substrate.id_substrate, minesota_code, name FROM substrate
  INNER JOIN complex_substrate ON complex_substrate.id_substrate=substrate.id_substrate
  INNER JOIN complex ON complex.id_complex=complex_substrate.id_complex
  WHERE activity='REG';
• *RegTransbase r20120406* [2]:
  The SQL request used to create this dataset is:
  SELECT DISTINCT a.pmid, e.name, r.name
  FROM regulator2effectors AS re
  INNER JOIN exp2effectors AS ee ON ee.effector_guid=re.effector_guid
  INNER JOIN dict_effectors AS e ON e.effector_guid=ee.effector_guid
  INNER JOIN regulators AS r ON r.regulator_guid=re.regulator_guid
  INNER JOIN articles AS a ON a.art_guid=ee.art_guid
  ORDER BY e.name;
  RegTransbase was not maintained anymore at the time of writing of this manuscript.
• *RegulonDB v9.0* [3]:
  The SQL request used to create this dataset is:
  SELECT c.conformation_id, c.final_state, e.effector_id, e.effector_name, tf.transcription_factor_id, tf.transcription_factor_name, p.reference_id, xdb.external_db_name
  FROM effector AS e
  INNER JOIN conformation_effector_link AS mm_ce ON mm_ce.effector_id=e.effector_id
  LEFT JOIN conformation AS c ON c.conformation_id=mm_ce.conformation_id
  LEFT JOIN transcription_factor AS tf ON tf.transcription_factor_id=c.transcription_factor_id
  LEFT JOIN object_ev_method_pub_link AS x ON x.object_id=c.conformation_id OR x.object_id=tf.transcription_factor_id OR x.object_id=e.effector_id
  LEFT JOIN publication AS p ON p.publication_id=x.publication_id
  LEFT JOIN external_db AS xdb ON xdb.external_db_id=p.external_db_id
  WHERE c.interaction_type IS Null OR c.interaction_type!='Covalent';
• *RegPrecise v4.0* [4]:
  The RegPrecise website was accessed (version v4.0) and all relevant data was extracted from the effector pages of the website.
• *Sigmol v20170216* [5]:
  Sigmol was accessed on 16/02/2017 and all effector data was retrieved from the unique *Quorum Sensing Signaling Molecule* page. In the “detected by” column, we provide the class of signaling compounds the compound belongs to. The comment field reads ‘Extracted from Sigmol v20170216 – Uniq_QSSM_“number”’.

### 2.1 Data overview

In Table 1 are presented some characteristics of each data source: number of compounds without a structure from this source, total number of compounds with a structure from this source and number of compounds with a structure found only in this source. The last column in particular shows that around half the compounds are found in more than one data source.

**Table 1:** Contribution of each data source.

**Source**	**Compounds without structure**	**Compounds with structure**	**Unique compounds with structure**
RegPrecise	136	418	73
BioNemo	5	499	8
RegTransBase	683	2057	63
RegulonDB	12	245	23
Sigmol	2	175	135
ACS Synthetic Biology	44	287	73
**All sources**	**882**	**3681**	**729**

The first column contains the data source, the second column the number of compounds found without a structure in that source, the third column the number of compounds with a structure (InChI) and the last column the number of compounds with a structure found only in that source.

**Fig. 1:**
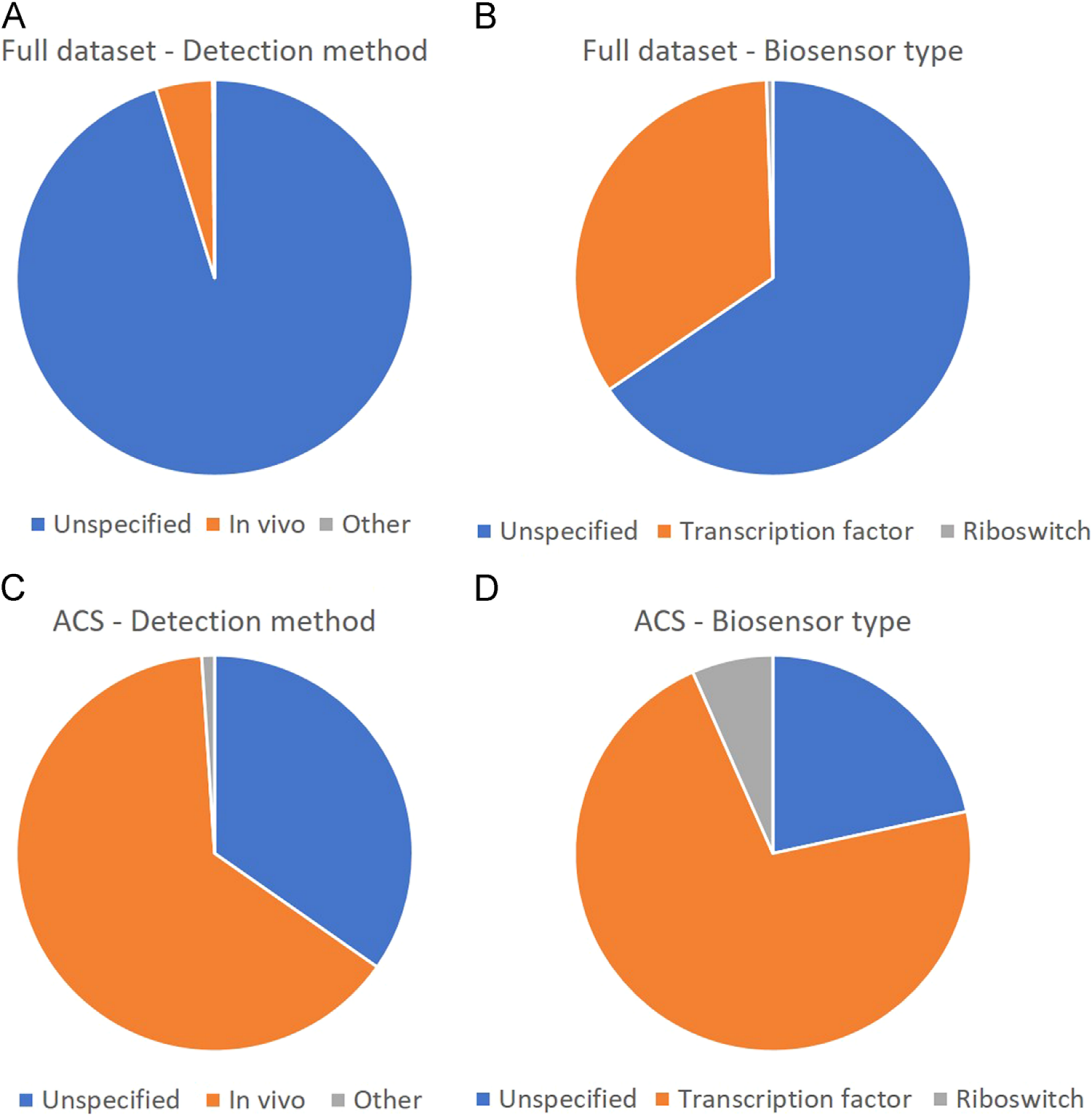
Type of experiment and biosensor type in the full dataset and the manually curated dataset. A: Full dataset – detection method. B: Full dataset – biosensor type. C: ACS dataset – detection method. D: ACS dataset – biosensor type. A and C: other in detection method corresponds to *in silico, in vivo* and *cell-free* detections. C and D: ACS dataset is the dataset obtained from manual curation of ACS Synthetic Biology with compounds that have available structures.

Fig. 1 shows the repartition of the type of experiment (*in vivo*, unspecified or other), as well as the repartition of Biosensor type (Transcription factor, riboswitch or unspecified) in the full dataset and the manually curated dataset from ACS Synthetic Biology.
